# Association of 24 h Behavior Rhythm with Non-Alcoholic Fatty Liver Disease among American Adults with Overweight/Obesity

**DOI:** 10.3390/nu15092101

**Published:** 2023-04-27

**Authors:** Wenbo Gu, Tianshu Han, Changhao Sun

**Affiliations:** Department of Nutrition and Food Hygiene, The National Key Discipline, School of Public Health, Harbin Medical University, Harbin 150081, China; wenbogu@hrbmu.edu.cn (W.G.); snowcalendar@126.com (T.H.)

**Keywords:** non-alcoholic fatty liver disease, activity–rest rhythm, feeding–fasting rhythm, obesity

## Abstract

Emerging evidence suggests that in addition to metabolic, genetic and environmental factors, circadian rhythm also plays a role in non-alcoholic fatty liver disease (NAFLD). This study aimed to explore the association of 24 h behavior rhythm (activity–rest and feeding–fasting rhythm) with NAFLD. A total of 4502 adult participants with overweight/obesity from the National Health and Nutrition Examination Survey (NHANES) 2011–2014 were included in the current study. The behavior rhythm indices were calculated and divided into quintiles for logistic regression models. Compared to those in the lowest quintile, participants in the highest quintile of relative amplitude (RA) had a lower risk of NAFLD (OR = 0.71, 95% CI, 0.55–0.91); participants in the highest quintile of the average activity of the least active continuous 5 h period (L5) were associated with a higher risk of NAFLD (OR = 1.35, 95% CI, 1.07–1.71). Additionally, participants in the highest quintile of fasting duration and feeding rhythm score were associated with a lower risk of NAFLD relative to those in the lowest quintile (OR = 0.76, 95% CI, 0.59–0.98 for fasting duration, OR = 0.74, 95% CI, 0.58–0.95 for feeding rhythm score). The associations were stronger among participants with obesity. No significant associations were found in the relationship of other behavior rhythm indices with NAFLD. This study indicated a significant association of 24 h behavior rhythm with NAFLD among American adults with overweight/obesity.

## 1. Introduction

The prevalence of non-alcoholic fatty liver disease (NAFLD) is high among populations with obesity or type 2 diabetes, though its cause is still unknown [1]. Epidemiological evidence has suggested that inadequate eating habits or unhealthy lifestyle can play a vital role in the development of NAFLD [2].

In response to the Earth’s rotation, organisms have evolved under the anticipated daily cycle of light and darkness and formed daily behavior patterns, such as 24 h activity/rest and feeding/fasting cycles, to adapt to these predictable changes in the environment [3,4]. These daily behavior patterns, together with light, which is the dominant zeitgeber for photosensitive clocks, are profound external clues that can entrain (or reset) the endogenous rhythms [5,6]. A small cohort study conducted in the laboratory found that participants suffering from recurring 28 h ‘day’ or shiftwork presented circadian misalignment on metabolic fitness, such as a decreased level of leptin and elevated level of glucose in circulation [7]. Other cross-sectional studies demonstrated that an aberrant activity–rest rhythm links with general and abdominal obesity [8], impaired glucose homeostasis [9,10] and increased white blood-cell-based inflammatory indices [11]. In recent years, time-restricted feeding (TRF) has begun to attract attention for its role in prevention of metabolic diseases such as obesity, glucose intolerance, leptin resistance and hepatic steatosis [12,13]. Studies have found that administration of melatonin (a hormone selected by the pineal gland that makes a critical contribution to circadian variation) could improve liver function and attenuate the course of NAFLD [14,15]. The liver, which is a critical organ that maintains metabolic homeostasis in the body [16], may be affected by these daily behavior patterns. However, to date, the link between daily behavior patterns and liver health has been poorly investigated; thus, we hypothesized the association of 24 h behavior rhythm (which includes activity–rest and feeding–fasting rhythm) with NAFLD in a population with overweight/obesity.

## 2. Materials and Methods

### 2.1. Study Population

This cross-sectional study aimed to explore the association of 24 h behavior rhythm with NAFLD among American adults with overweight/obesity. We selected adult participants (age ≥ 18) with overweight/obesity from the National Health and Nutrition Examination Survey (NHANES) 2011–2014, which is a multistage and stratified study designed to represent the national population in the United States. Overweight/obesity was defined as body mass index (BMI) ≥ 25. We excluded participants with high alcohol consumption (≥20 g/day for male and ≥10 g/day for female), and those who had positive serum hepatitis B surface antigen or positive serum hepatitis C antibody. Participants with missing values of covariates were also excluded. The detailed flowchart for participant selection is presented in Figure S1. Overall, a total of 4502 participants were included. All protocols were approved by the National Center for Health Statistics Research Ethics Review Board, and participants were provided informed consent.

### 2.2. Activity–Rest Rhythm Indices

The activity–rest rhythm indices were calculated as our previous study demonstrated [17]. Briefly, participants wore an accelerometer (GT3X+, ActiGraph Corporation, Pensacola, FL, USA) on their nondominant wrist for 7 continuous days. Data were imputed using the ‘accelmissing’ package and activity–rest rhythm indices were calculated with the ‘nparACT’ package. The output variables included the following: (1) Interdaily stability (IS) quantifies the stability of activity–rest rhythm and a value closer to 1 indicates a stronger conformity to a 24 h activity cosine model. (2) Intradaily variability (IV) quantifies the fragmentation of the activity–rest rhythm and a value closer to 2 indicates more “up and down” rather than maintaining consistent activity or rest. (3) The average activity of the most active continuous 10 h period (M10). (4) The average activity of the least active continuous 5 h period (L5). (5) Relative amplitude (RA) reflects the amplitude of activity and rest pattern and is calculated using the following equation: RA = (M10 − L5)/(M10 + L5). (6) Onset time of M10 (M10 start time) and onset time of L5 (L5 start time) provide information on the start time of activity and rest, respectively.

### 2.3. Feeding–Fasting Rhythm Indices

Timing for meals was collected by responding to the question “What time did you begin to eat/drink the meal/food?”. Amount of total feeding period = timing for the last meal − timing for the first meal; therefore, fasting duration = 24 h − amount of total feeding period. Moreover, in order to explore whether one’s feeding behavior fits their activity–rest pattern, we determined a feeding rhythm score to reflect the alignment extent. Firstly, we calculated the overlapping amounts of total feeding period with the most active 10 h period (M10 start time ~ M10 end time) and with the least active 5 h period (L5 start time ~ L5 end time). Then, the two overlapping amounts were divided into the amount of total feeding period to calculate the proportions of feeding period occurring during the active period and the inactive period in the total feeding period, respectively. Researchers have found that feeding during the active period is beneficial for health, while feeding during the inactive period shows opposite effects [12,18]. Therefore, the final feeding rhythm score was calculated as follows: feeding rhythm score = (overlapping amount of total feeding period with the most active 10 h period/amount of total feeding period) − (overlapping amount of total feeding period with the least active 5 h period/amount of total feeding period). For example, if one’s first and last meal were consumed at 07:00 and 19:00; the M10 start time and M10 end time were 09:00 and 19:00; the L5 start time and L5 end time were 03:00 and 08:00, then the feeding rhythm score is 0.75 (i.e., [(19 − 9)/(19 − 7)] − [(8 − 7)/(19 − 7)] = 0.75). A higher value of feeding rhythm score (close to 1) indicates that feeding behavior is more aligned with the activity–rest pattern.

### 2.4. NAFLD Assessment

Serum alanine aminotransferase (ALT) is a widely used biomarker for liver injury, and its elevation can be a diagnostic biomarker for NAFLD after excluding the effects of alcohol or viral hepatitis [19]. Serum ALT concentrations (U/L) were measured using a Beckman UniCel DxC 800 Synchron (Beckman Coulter, Inc., Brea, CA, USA) with a kinetic rate method. NAFLD status was defined as serum ALT > 30 U/L for males and > 19 U/L for females in the absence of elevated serum ALT levels caused by other reasons such as hepatitis B or C, and excessive alcohol intake.

### 2.5. Covariates 

Potential covariates included age, gender (male/female), race (Mexican American, other Hispanic, non-Hispanic White, non-Hispanic Black or other races), BMI (calculated as weight (kg)/height (m)^2^), income (<USD 20,000, USD 20,000–45,000, USD 45,000–75,000, USD 75,000–100,000 or >USD 100,000), education level (<9th grade, 9–11th grade, high school graduate, GED or equivalent, some college or AA degree, college graduate or above), current smoking status (yes/no), current drinking status (yes/no), regular exercise (yes/no), energy (kcal/d), self-reported of diabetes, hypertension and hypercholesterolemia.

### 2.6. Statistical Analyses

Demographic characteristics, lifestyle habits and chronic-disease status by NAFLD status were presented as weighted means with standard deviation (SD) for continuous variables and weighted percentages with SD for categorical variables. *p*-values were calculated using general linear models adjusted for age for continuous variables and chi-squared test for categorical variables between the non-NAFLD and NAFLD group.

Binary logistic regression models were developed to examine the association of 24 h behavior rhythm with NAFLD and odds ratios (ORs) with 95% confidence intervals (CIs) were calculated. The indices of behavior rhythm were classified into quintiles, with the lowest quintile used as the referent group. The statistical analyses were performed using R software (version 4.1.2), and incorporated sample weights according to NHANES analytic guidelines to ensure the nationally representative estimates. All tests were two-tailed, and *p* < 0.05 was considered statistically significant.

### 2.7. Sensitivity Analyses

Several sensitivity analyses were carried out to test the robustness of our findings. Firstly, considering the possible effect of diet on NAFLD [20], we further adjusted for the alternative healthy eating index (AHEI) based on the fully adjusted model. Secondly, chronic diseases, such as cancer and cardiovascular diseases (CVDs, including congestive heart failure, coronary heart disease, angina, heart attack and stroke) were additionally adjusted to exclude their possible interrelationships [21]. Thirdly, a sensitivity analysis was performed using unweighted data to confirm the stability of the weighted estimates. Finally, we performed subgroup analyses according to smoking status (current smoker and current non-smoker), drinking status (current drinker and current non-drinker) and exercise status (regular exercise and non-regular exercise).

## 3. Results

### 3.1. Baseline Characteristics

The mean age of the participants was 49.18 years with 51.26% being female and 66.07% Non-Hispanic White. Of the total 4502 participants, 1676 (37.23%) were identified as suspected NAFLD cases. Participants with NAFLD were more likely to be female and relatively young, and had a higher BMI, lower energy intake, higher L5 and lower fasting duration, compared with those with non-NAFLD. Other details of the baseline characteristics of the population are summarized in Table 1.

### 3.2. Association of 24 h Behavior Rhythm Indices with NAFLD among Participants with Overweight/Obesity

**Figure 1:**
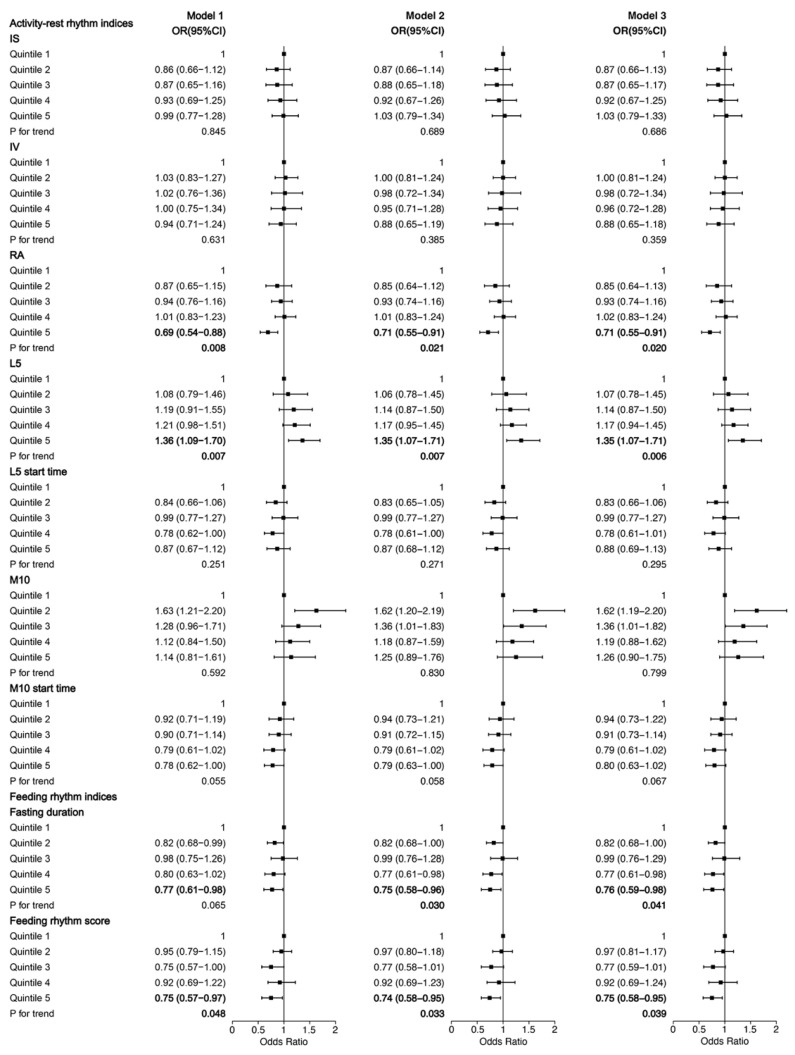
Association of 24 h behavior rhythm indices with NAFLD among participants with overweight/obesity. Model 1 was adjusted for age, gender and race; Model 2 was additionally adjusted for BMI, education level, income, current smoking status, current drinking status, regular exercise and energy; Model 3 was further adjusted for self-reported diabetes, hypertension and hypercholesterolemia. BMI, body mass index; Q, quartile; IS, interdaily stability; IV, intradaily variability; RA, relative amplitude; L5, average activity of the least active continuous 5 h period; M10, average activity of the most active continuous 10 h period; OR, odds ratio; CI, confidence interval.

The association of 24 h behavior rhythm indices with NAFLD among participants with overweight/obesity is shown in Figure 1. Compared with participants in quintile 1 of RA and L5, those in quintile 5 had a lower risk (OR = 0.69, 95% CI, 0.54–0.88) and a higher risk (OR = 1.36, 95% CI, 1.09–1.70) of NAFLD, respectively, when adjusted for age, gender and race in Model 1. When further adjusted for other demographic, lifestyle habits and disease confounders, participants in quintile 5 of RA and L5 were still associated with NAFLD in Model 2 and Model 3 (ORModel2 = 0.71, 95% CI, 0.55–0.91 for RA, ORModel2 = 1.35, 95% CI, 1.07–1.71 for L5); (ORModel3 = 0.71, 95% CI, 0.55–0.91 for RA, ORModel3 = 1.35, 95% CI, 1.07–1.71 for L5). Additionally, compared with participants in quintile 1 of fasting duration and feeding rhythm score, those in quintile 5 had a lower risk of NAFLD (OR = 0.77, 95% CI, 0.61–0.98 for fasting duration, OR = 0.75, 95% CI, 0.57–0.97 for feeding rhythm score) when adjusted for age, gender and race in Model 1. Similarly, when adjusted for other confounders, participants in quintile 5 of feeding rhythm score and fasting duration were still associated with a lower risk of NAFLD in Model 2 and Model 3 among participants with overweight/obesity (ORModel2 = 0.75, 95% CI, 0.58–0.96 for fasting duration, ORModel2 = 0.74, 95% CI, 0.58–0.95 for feeding rhythm score); (ORModel3 = 0.76, 95% CI, 0.59–0.98 for fasting duration, ORModel3 = 0.74, 95% CI, 0.58–0.95 for feeding rhythm score).

### 3.3. Association of 24 h Behavior Rhythm Indices with NAFLD among Participants with Obesity 

**Figure 2:**
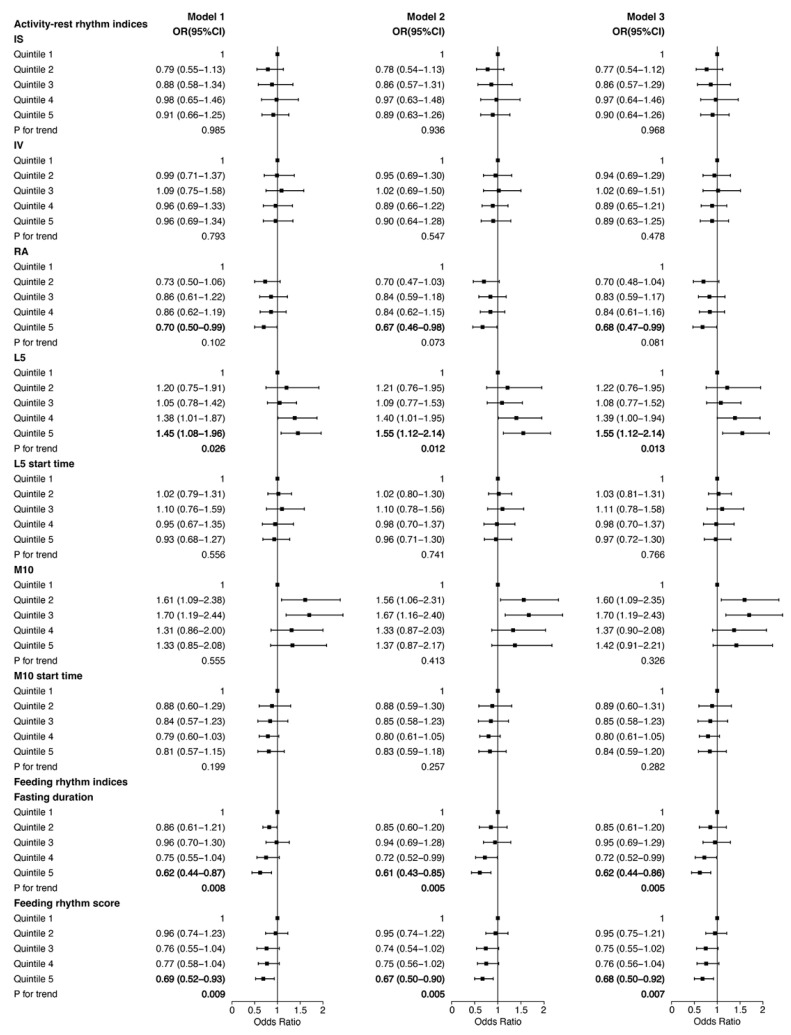
Association of 24 h behavior rhythm indices with NAFLD among participants with obesity. Model 1 was adjusted for age, gender and race; Model 2 was additionally adjusted for BMI, education level, income, current smoking status, current drinking status, regular exercise and energy; Model 3 was further adjusted for self-reported diabetes, hypertension and hypercholesterolemia. BMI, body mass index; Q, quartile; IS, interdaily stability; IV, intradaily variability; RA, relative amplitude; L5, average activity of the least active continuous 5 h period; M10, average activity of the most active continuous 10 h period; OR, odds ratio; CI, confidence interval.

In order to examine whether the association of 24 h behavior rhythm indices with NAFLD is more obvious in those with higher BMI, we performed the same analyses among participants with obesity (defined as BMI ≥ 30) and the results are shown in Figure 2. The ORs of RA were similar to those in participants with overweight/obesity; while participants in quintile 5 of L5 had significantly higher ORs for NAFLD among participants with obesity compared with those in participants overweight/obesity. In Model 1, when adjusted for age, gender and race, participants in quintile 5 of L5 had an OR of 1.45 (95% CI, 1.08–1.96) among participants with obesity. When further adjusted for other demographic, lifestyle habits and disease confounders, participants in quintile 5 of L5 were still associated with a higher risk of NAFLD in Model 2 and Model 3 among participants with obesity (ORModel2 = 1.55, 95% CI, 1.12–2.14, ORModel3 = 1.55, 95% CI, 1.12–2.14). Additionally, the associations between feeding–fasting rhythm and NAFLD were also stronger among participants with obesity than those in participants with overweight/obesity. In Model 1, when adjusted for age, gender and race, participants in quintile 5 of fasting duration and feeding rhythm score had an OR of 0.62 (95% CI, 0.44–0.87) and 0.69 (95% CI, 0.52–0.93) for NAFLD, respectively. When adjusted for other confounders, participants in quintile 5 of fasting duration and feeding rhythm score were still associated with a lower risk of NAFLD among participants with obesity (ORModel2 = 0.61, 95% CI, 0.43–0.85 for fasting duration, ORModel2 = 0.67, 95% CI, 0.50–0.90 for feeding rhythm score); (ORModel3 = 0.62, 95% CI, 0.44–0.86 for fasting duration, ORModel3 = 0.68, 95% CI, 0.50–0.92 for feeding rhythm score).

### 3.4. Sensitivity Analyses

The associations of 24 h behavior rhythm with NAFLD remained consistent when considering additional confounders including AHEI, cancer and cardiovascular diseases, or using unweighted data to repeat the analyses, and were stable among current drinkers, current smokers and participants with non-regular exercise. Contrarily, no associations of 24 h behavior rhythm indices with NAFLD were found among current non-smokers, current non-drinkers or participants with regular exercise. The details of the sensitivity analyses are shown in Figures S2–S6. 

## 4. Discussion

### 4.1. Summary of This Study

The current study investigated whether the 24 h behavior rhythm, composed of activity–rest and feeding–fasting rhythm, was associated with NAFLD among adults with overweight/obesity in the United States. In this study, patients with NAFLD had a significantly higher BMI than healthy individuals. This finding suggests that these patients may consciously manage their energy intake, control their body weight, improve their dietary habits, and increase their physical activity. As a result, participants with NAFLD may have a lower energy intake and a lower smoking rate, and the prevalence of diabetes, hypertension and hypercholesterolemia among these individuals may be similar to or slightly higher than that of the non-NAFLD population. We found that a higher RA was associated with a 29% lower risk of NAFLD, and a higher L5 was associated with a 35% higher risk of NAFLD, whereas no significant associations were found in the relationship of IS, IV or M10 with NAFLD. Moreover, a higher fasting duration was found associated with a 24% lower risk of NAFLD. Specifically, we defined a feeding rhythm score that reflects whether and how one’s feeding behavior is aligned with the activity–rest pattern. We found that a higher feeding rhythm score was associated with a 25% lower risk of NAFLD. In addition, we found that the associations between some behavior rhythm indices (L5, fasting duration and feeding rhythm score) and NAFLD were stronger among participants with obesity. Our results were stable when additionally adjusted for AHEI, cancer and CVDs, or using unweighted data to repeat the analyses. The associations also existed in subgroups of current smokers, current drinkers and participants with non-regular exercise.

### 4.2. Activity–Rest Rhythm and NAFLD

Studies have provided evidence that individuals with obesity and insulin resistance show a smaller RA, lower IS and higher IV [22], which can cause misaligned endogenous rhythms through providing feedback to the central clock [22,23]. The impaired circadian clock further interacts with lipid metabolic outcomes by affecting the rhythmicity of hormone secretion, gut microbiome and energy homeostasis [24]. The studies above supported our finding that, in participants with overweight/obesity, a strong circadian rhythm (identified by a high RA) may function as a positive regulator of lipid metabolism and improve the course of NAFLD. Although previous studies indicated that lower IS and higher IV were associated with elevated levels of total cholesterol and C-reactive protein [25,26], which play a crucial role in the pathogenesis of NAFLD [27,28], their effects on NAFLD may be masked by the adverse effects of overweight and obesity in the current study. Moreover, in our study, a higher L5, which reflects a more fragmented and disrupted sleep, was found associated with a higher risk of NAFLD. An observational study directly supported our finding, revealing a correlation between poor sleep quality and an increased risk of NAFLD [29]. Another population study suggested that poor sleep quality has a 20% prediction of the variability in liver stiffness [30]. Mechanism studies have found that poor sleep quality may cause NAFLD through several converging pathways, including insulin resistance, adipose dysfunction, weight gain and systemic inflammation [31].

### 4.3. Feeding–Fasting Rhythm and NAFLD

Numerous studies have revealed that having a long fasting duration and eating at the right time are beneficial for health and can prolong the lifespan in humans and other species [32,33,34]. In the current study, we found that high fasting duration and feeding rhythm score were associated with a lower risk of NAFLD, which could be explained by the existing studies. A pilot study found that in overweight individuals, reducing the daily eating duration contributes to weight loss [35], which is a first-line intervention for NAFLD [36]. Another animal study showed that TRF could reduce the accumulation of hepatic lipids and enhance cellular response to metabolic stress in mice lacking a circadian clock [37]. Furthermore, a recent mechanism study demonstrated that rhythmic creatine-mediated thermogenesis played an essential role in TRF-induced metabolic benefits [38]. On the other hand, eating at the right time, which means restricting feeding to the usual active period, is equally important in synergistic interactions between the feeding/fasting signals and circadian rhythm. It has been reported that feeding rhythms have an essential influence on the biodiversity and balance of intestinal microbiota, such as probiotics [39], and the supplement of probiotics was proved to improve hepatic enzymes and decrease BMI in NAFLD patients with obesity and overweight [40]. A mistimed eating time can reset many circadian clocks in peripheral organs and brain, and can cause detrimental effects on metabolic health [41]. Cross-sectional studies suggested that high consumption of calories in the evening or night was associated with increased body fat and higher risk of overweight and obesity [18,42]. An animal study also indicated that mice fed in light phase (which is inactive phase for mice) showed an altered energy balance and dyssynchrony between metabolically active organs [43]. Conversely, mice restrictedly fed in nighttime (which is active phase for mice) showed an altered phase of triglyceride accumulation and decreased hepatic triglyceride levels [44], which provided evidence to support our finding.

### 4.4. Strengths and Limitations

Our study has several strengths. Firstly, we took good advantage of high-quality multi-day objective actigraphy data and dietary data from the nationally representative study sample in the United States, and innovatively combined these two data types to produce a feeding rhythm score that reflects how one’s feeding/fasting cycle fits their activity/rest cycle. Secondly, this study effectively demonstrated the association between 24 h behavior rhythm and NAFLD in adults with overweight/obesity, and the results were stable and robust after additionally adjustment for AHEI, cancer and CVDs. Thirdly, we performed the same analyses among participants with obesity, current drinkers, current smokers and participants with non-regular exercise, and the consistent results indicated that our findings were applicable to those with a high risk of NAFLD.

Despite all the efforts, we recognized that our study had certain limitations. Firstly, we failed to establish the temporal/causal relationship due to the cross-sectional nature of this study. Secondly, although ALT is commonly used as a marker to screen for suspected NAFLD, there are conditions that can cause ALT levels to be elevated due to other factors, such as viral hepatitis, autoimmune hepatitis, alcohol consumption and certain medications. Therefore, further testing is needed to confirm a diagnosis of NAFLD. Thirdly, despite considering a series of confounders, there is possibility that residual confounders may still exist in the associations between behavior rhythm and serum alanine aminotransferase levels. Further studies are needed to explore the roles of those unmeasured variables that affect both behavior rhythm and NAFLD. Fourthly, the dietary data were collected on a random day of the week, while studies have found that eating patterns are erratic and vary between weekdays and weekends [35]. Fifthly, information on shift workers was not included in this dataset, so we are unable to evaluate the role of shift work status in the current study. Finally, participants were all adults with overweight/obesity in the United States; thus, these findings need to be confirmed in other populations and countries. 

### 4.5. Conclusions

This study indicated a significant association of 24 h behavior rhythm with NAFLD among adults with overweight/obesity in the United States. Our study can shed new light on the prevention of NAFLD in a high-risk population. 

## Figures and Tables

**Table 1:** Characteristics of the participants by non-alcoholic fatty liver disease (NAFLD) status.

Characteristics	Total	Non-NAFLD	NAFLD	*p*-Value
	(n = 4502)	(n = 2826)	(n = 1676)	
Age, years	49.18 (0.48)	50.39 (0.55)	47.18 (0.52)	<0.001
Female, (%)	51.26 (0.89)	46.42 (1.02)	59.21 (1.45)	<0.001
Non-Hispanic White, (%)	66.07 (2.89)	67.53 (2.30)	63.68 (3.14)	<0.001
BMI, kg/m^2^	32.26 (0.19)	31.67 (0.19)	33.23 (0.27)	<0.001
>USD 100,000 annual household income, (%)	19.71 (1.57)	19.77 (1.85)	19.61 (1.73)	0.729
College graduate or above, (%)	25.11 (1.28)	26.16 (1.43)	23.39 (1.64)	0.106
Current smoking, (%)	17.53 (0.93)	18.87 (1.02)	15.34 (1.46)	0.044
Current drinking, (%)	69.66 (1.63)	70.87 (1.26)	67.67 (2.71)	0.142
Regular exercise, (%)	24.46 (0.83)	24.18 (1.00)	24.93 (1.24)	0.614
Energy, kcal	2048.39 (18.16)	2061.00 (16.98)	2027.65 (27.41)	0.007
Self-reported diabetes, (%)	13.96 (0.59)	13.61 (0.67)	14.55 (1.23)	0.354
Self-reported hypertension, (%)	39.22 (1.16)	39.19 (1.33)	39.26 (2.06)	0.976
Self-reported hypercholesterolemia, (%)	39.97 (1.09)	40.01 (1.27)	39.91 (1.53)	0.929
AHEI	53.37 (0.37)	53.62 (0.32)	52.96 (0.59)	0.125
Cancer, (%)	10.70 (0.63)	10.62 (1.02)	10.84 (0.87)	0.882
Cardiovascular diseases, (%)	10.17 (0.52)	11.59 (0.57)	7.84 (0.67)	<0.001
IS	0.58 (0.00)	0.58 (0.00)	0.58 (0.00)	0.868
IV	0.70 (0.01)	0.71 (0.01)	0.69 (0.01)	0.242
RA	0.84 (0.00)	0.85 (0.00)	0.84 (0.00)	0.053
L5	1.20 (0.02)	1.16 (0.02)	1.28 (0.03)	0.029
L5 start time, h:m:s	00:45:56 (00:02:36)	00:45:18 (00:03:16)	00:46:58 (00:03:13)	0.596
M10	14.13 (0.09)	13.99 (0.11)	14.34 (0.13)	0.576
M10 start time, h:m:s	09:18:11 (00:02:53)	09:17:17 (00:03:54)	09:19:40 (00:05:04)	0.365
Fasting duration, hour	11.30 (0.05)	11.35 (0.06)	11.22 (0.08)	0.042
Feeding rhythm score	0.73 (0.00)	0.73 (0.00)	0.72 (0.01)	0.072

Continuous and categorical variables are presented as weighted means (SD) and weighted percentages (SD), respectively. BMI, body mass index. AHEI, alternative healthy eating index. *p*-values were calculated using general linear models adjusted for age for continuous variables and chi-squared test for categorical variables between non-NAFLD and NAFLD groups.

## Data Availability

The data presented in this study are openly available in the U.S. National Health and Nutrition Examination Survey at [https://www.cdc.gov/nchs/nhanes/index.htm] (accessed on 16 April 2023), reference number 2011–2014.

## Supplementary Materials

The following supporting information can be downloaded at: https://www.mdpi.com/article/10.3390/nu15092101/s1, Figure S1: Flowchart for participant selection. ALT, alanine aminotransferase; BMI, body mass index. Figure S2: Association of 24 h behavior rhythm indices with NAFLD when additionally adjusted for AHEI, cancer and CVDs among participants with overweight/obesity. BMI, body mass index; Q, quartile; IS, interdaily stability; IV, intradaily variability; RA, relative amplitude; L5, average activity of the least active continuous 5 h period; M10, average activity of the most active continuous 10 h period. Figure S3: Association of 24 h behavior rhythm indices with NAFLD among participants with overweight/obesity when using unweighted data. Model 1 was adjusted for age, gender and race; Model 2 was additionally adjusted for BMI, education level, income, current smoking status, current drinking status, regular exercise and energy; Model 3 was further adjusted for self-reported diabetes, hypertension and hypercholesterolemia. BMI, body mass index; Q, quartile; IS, interdaily stability; IV, intradaily variability; RA, relative amplitude; L5, average activity of the least active continuous 5 h period; M10, average activity of the most active continuous 10 h period. Figure S4: Association of 24 h behavior rhythm indices with NAFLD among current drinkers with overweight/obesity. Model 1 was adjusted for age, gender and race; Model 2 was additionally adjusted for BMI, education level, income, current smoking status, regular exercise and energy; Model 3 was further adjusted for self-reported diabetes, hypertension and hypercholesterolemia. BMI, body mass index; Q, quartile; IS, interdaily stability; IV, intradaily variability; RA, relative amplitude; L5, average activity of the least active continuous 5 h period; M10, average activity of the most active continuous 10 h period. Figure S5: Association of 24 h behavior rhythm indices with NAFLD among current smokers with overweight/obesity. Model 1 was adjusted for age, gender and race; Model 2 was additionally adjusted for BMI, education level, income, current drinking status, regular exercise and energy; Model 3 was further adjusted for self-reported diabetes, hypertension and hypercholesterolemia. BMI, body mass index; Q, quartile; IS, interdaily stability; IV, intradaily variability; RA, relative amplitude; L5, average activity of the least active continuous 5 h period; M10, average activity of the most active continuous 10 h period. Figure S6: Association of 24 h behavior rhythm indices with NAFLD among participants with overweight/obesity and non-regular exercise. Model 1 was adjusted for age, gender and race; Model 2 was additionally adjusted for BMI, education level, income, current smoking status, current drinking status and energy; Model 3 was further adjusted for self-reported diabetes, hypertension and hypercholesterolemia. BMI, body mass index; Q, quartile; IS, interdaily stability; IV, intradaily variability; RA, relative amplitude; L5, average activity of the least active continuous 5 h period; M10, average activity of the most active continuous 10 h period.
