# Supplementary material for: Association of 24 h Behavior Rhythm with Non-Alcoholic Fatty Liver Disease among American Adults with Overweight/Obesity
Source: Nutrients. 2023 Apr 27;15(9):2101. doi: 10.3390/nu15092101 (PMC10180861; doi:10.3390/nu15092101)
Supplement: Supplementary file 1 [file nutrients-15-02101-s001.zip › nutrients-2346179-supplementary.pdf]

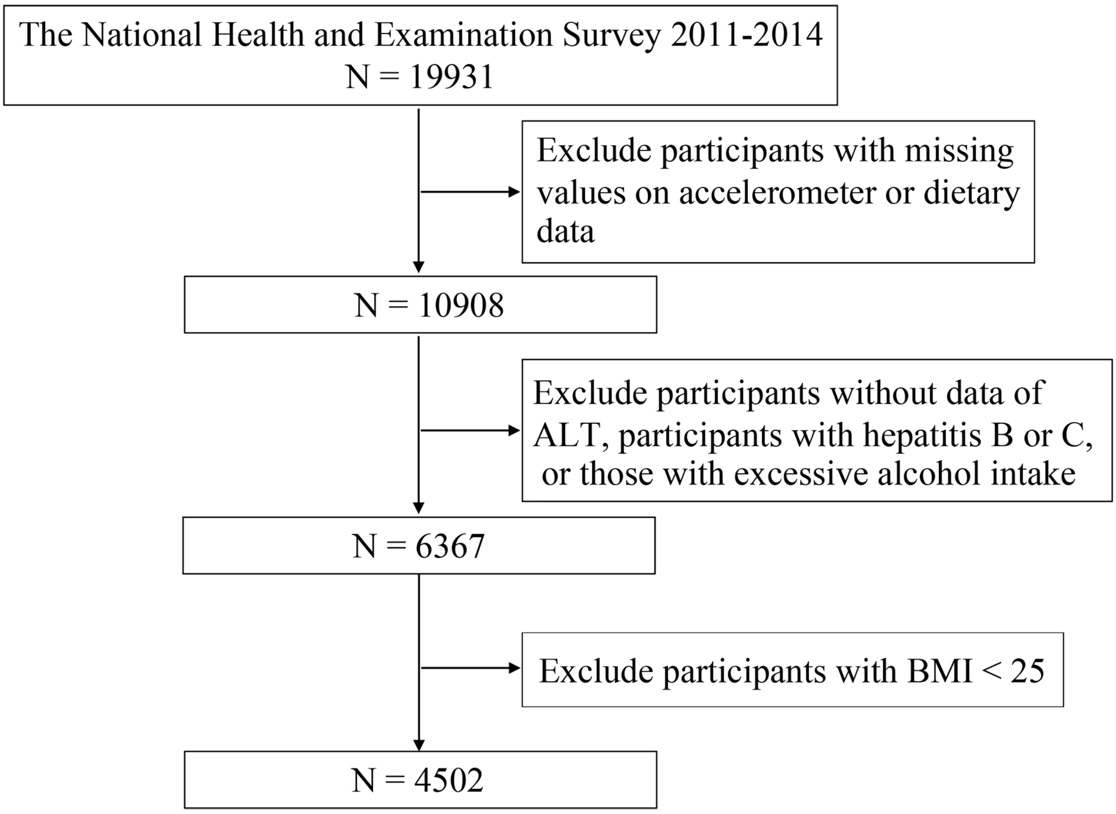

Figure S1. Flowchart for participant selection. ALT, alanine aminotransferase; BMI, body mass index.

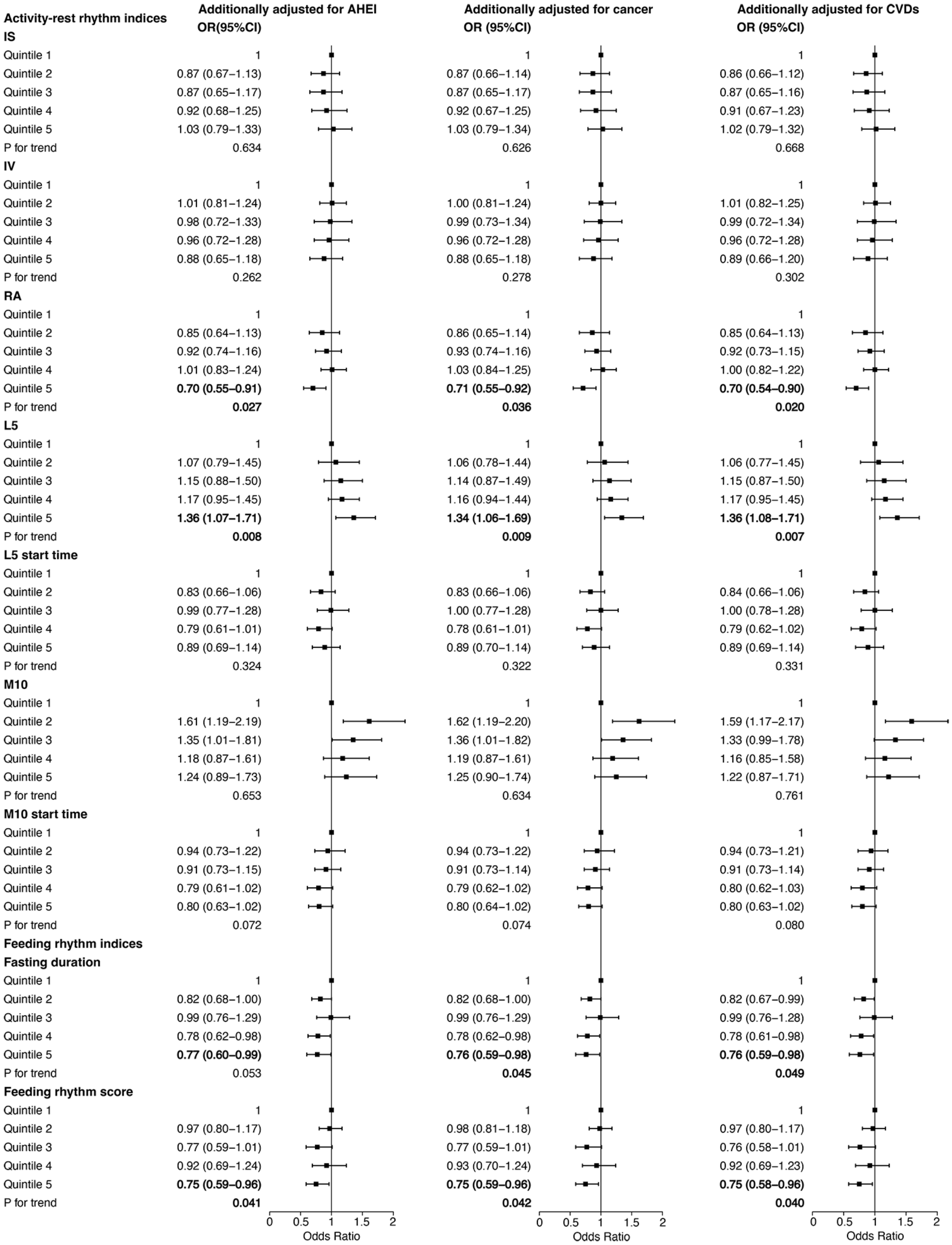

Figure S2. Association of 24 h behavior rhythm indices with NAFLD when additionally adjusted for AHEI, cancer and CVDs among participants with overweight/obesity. BMI, body mass index; Q, quartile; IS, interdaily stability; IV, intradaily variability; RA, relative amplitude; L5, average activity of the least active continuous 5 h period; M10, average activity of the most active continuous 10 h period.

Activity-rest rhythm indices	Model 1 OR (95%CI)	Model 2 OR (95%CI)	Model 3 OR (95%CI)
**IS**			
Quintile 1	1	1	1
Quintile 2	0.86 (0.70–1.05)	0.86 (0.71–1.05)	0.86 (0.71–1.06)
Quintile 3	0.91 (0.74–1.10)	0.91 (0.74–1.11)	0.91 (0.75–1.11)
Quintile 4	0.93 (0.76–1.13)	0.93 (0.76–1.14)	0.93 (0.76–1.13)
Quintile 5	0.99 (0.82–1.21)	1.03 (0.84–1.26)	1.03 (0.84–1.26)
P for trend	0.895	0.657	0.686
**IV**			
Quintile 1	1	1	1
Quintile 2	1.02 (0.84–1.24)	1.00 (0.82–1.21)	1.00 (0.82–1.22)
Quintile 3	1.00 (0.82–1.22)	0.96 (0.78–1.17)	0.96 (0.79–1.18)
Quintile 4	1.07 (0.88–1.30)	1.02 (0.84–1.25)	1.02 (0.84–1.25)
Quintile 5	0.93 (0.76–1.14)	0.87 (0.71–1.08)	0.88 (0.71–1.09)
P for trend	0.595	0.253	0.287
**RA**			
Quintile 1	1	1	1
Quintile 2	0.91 (0.75–1.11)	0.91 (0.74–1.11)	0.91 (0.75–1.11)
Quintile 3	0.91 (0.75–1.10)	0.91 (0.75–1.12)	0.92 (0.75–1.12)
Quintile 4	1.02 (0.83–1.25)	1.04 (0.84–1.27)	1.04 (0.84–1.28)
Quintile 5	**0.77 (0.64–0.94)**	**0.81 (0.66–0.98)**	**0.81 (0.66–0.98)**
P for trend	0.056	0.152	0.150
**L5**			
Quintile 1	1	1	1
Quintile 2	1.06 (0.87–1.29)	1.05 (0.86–1.27)	1.05 (0.86–1.27)
Quintile 3	1.06 (0.87–1.29)	1.03 (0.84–1.25)	1.03 (0.84–1.25)
Quintile 4	1.11 (0.91–1.35)	1.08 (0.88–1.32)	1.08 (0.88–1.32)
Quintile 5	**1.25 (1.03–1.52)**	1.22 (1.00–1.49)	**1.22 (1.01–1.49)**
P for trend	**0.018**	**0.035**	**0.037**
**L5 start time**			
Quintile 1	1	1	1
Quintile 2	0.94 (0.77–1.15)	0.94 (0.77–1.15)	0.95 (0.78–1.16)
Quintile 3	1.09 (0.90–1.33)	1.09 (0.90–1.33)	1.10 (0.90–1.34)
Quintile 4	0.96 (0.78–1.16)	0.96 (0.78–1.16)	0.96 (0.78–1.17)
Quintile 5	1.02 (0.83–1.24)	1.02 (0.83–1.25)	1.03 (0.84–1.26)
P for trend	0.844	0.814	0.747
**M10**			
Quintile 1	1	1	1
Quintile 2	1.25 (1.02–1.53)	1.25 (1.02–1.53)	1.26 (1.03–1.54)
Quintile 3	1.17 (0.95–1.44)	1.21 (0.99–1.49)	1.21 (0.98–1.49)
Quintile 4	1.10 (0.90–1.36)	1.15 (0.93–1.42)	1.15 (0.93–1.43)
Quintile 5	1.05 (0.85–1.30)	1.13 (0.91–1.41)	1.12 (0.90–1.40)
P for trend	0.777	0.607	0.654
**M10 start time**			
Quintile 1	1	1	1
Quintile 2	1.10 (0.90–1.33)	1.10 (0.90–1.34)	1.10 (0.91–1.34)
Quintile 3	0.99 (0.82–1.21)	0.99 (0.81–1.21)	0.99 (0.82–1.21)
Quintile 4	0.92 (0.76–1.13)	0.92 (0.75–1.12)	0.92 (0.76–1.13)
Quintile 5	0.95 (0.77–1.16)	0.95 (0.77–1.17)	0.96 (0.78–1.18)
P for trend	0.272	0.283	0.312
**Feeding rhythm indices**			
**Fasting duration**			
Quintile 1	1	1	1
Quintile 2	0.92 (0.76–1.12)	0.93 (0.76–1.13)	0.93 (0.76–1.13)
Quintile 3	0.99 (0.81–1.20)	0.98 (0.80–1.20)	0.98 (0.80–1.20)
Quintile 4	0.80 (0.66–0.98)	0.78 (0.64–0.96)	0.79 (0.65–0.97)
Quintile 5	**0.81 (0.66–0.98)**	**0.77 (0.63–0.95)**	**0.78 (0.64–0.96)**
P for trend	**0.012**	**0.004**	**0.006**
**Feeding rhythm score**			
Quintile 1	1	1	1
Quintile 2	0.99 (0.82–1.20)	1.01 (0.83–1.23)	1.01 (0.83–1.23)
Quintile 3	0.86 (0.71–1.05)	0.88 (0.72–1.07)	0.88 (0.73–1.07)
Quintile 4	0.95 (0.78–1.15)	0.95 (0.78–1.15)	0.95 (0.78–1.16)
Quintile 5	**0.78 (0.64–0.95)**	**0.78 (0.64–0.95)**	**0.78 (0.64–0.95)**
P for trend	**0.012**	**0.009**	**0.012**

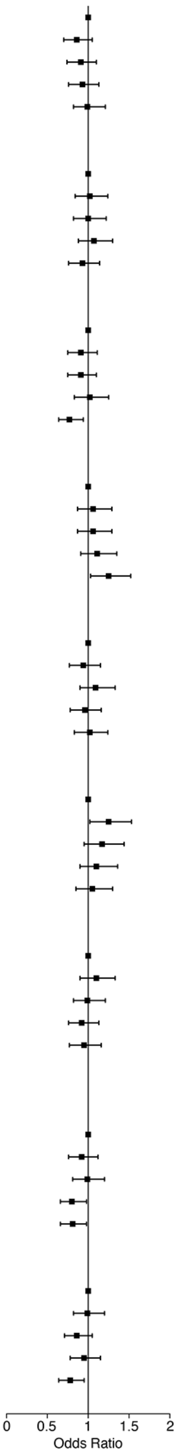

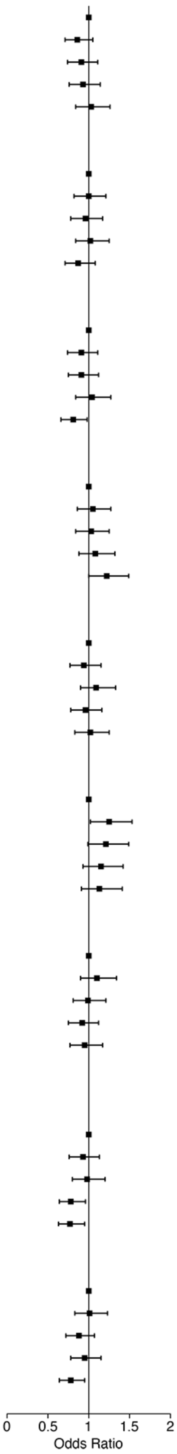

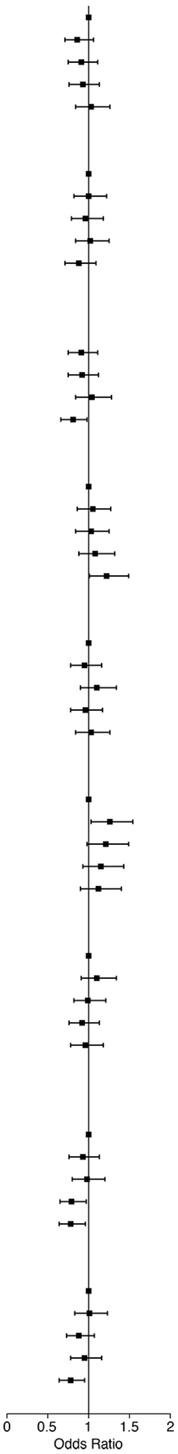

Figure S3. Association of 24 h behavior rhythm indices with NAFLD among participants with over-weight/obesity when using unweighted data. Model 1 was adjusted for age, gender and race; Model 2 was additionally adjusted for BMI, education level, income, current smoking status, current drinking status, regular exercise and energy; Model 3 was further adjusted for self-reported diabetes, hypertension and hypercholesterolemia. BMI, body mass index; Q, quartile; IS, interdaily stability; IV, intradaily variability; RA, relative amplitude; L5, average activity of the least active continuous 5 h period; M10, average activity of the most active continuous 10 h period.

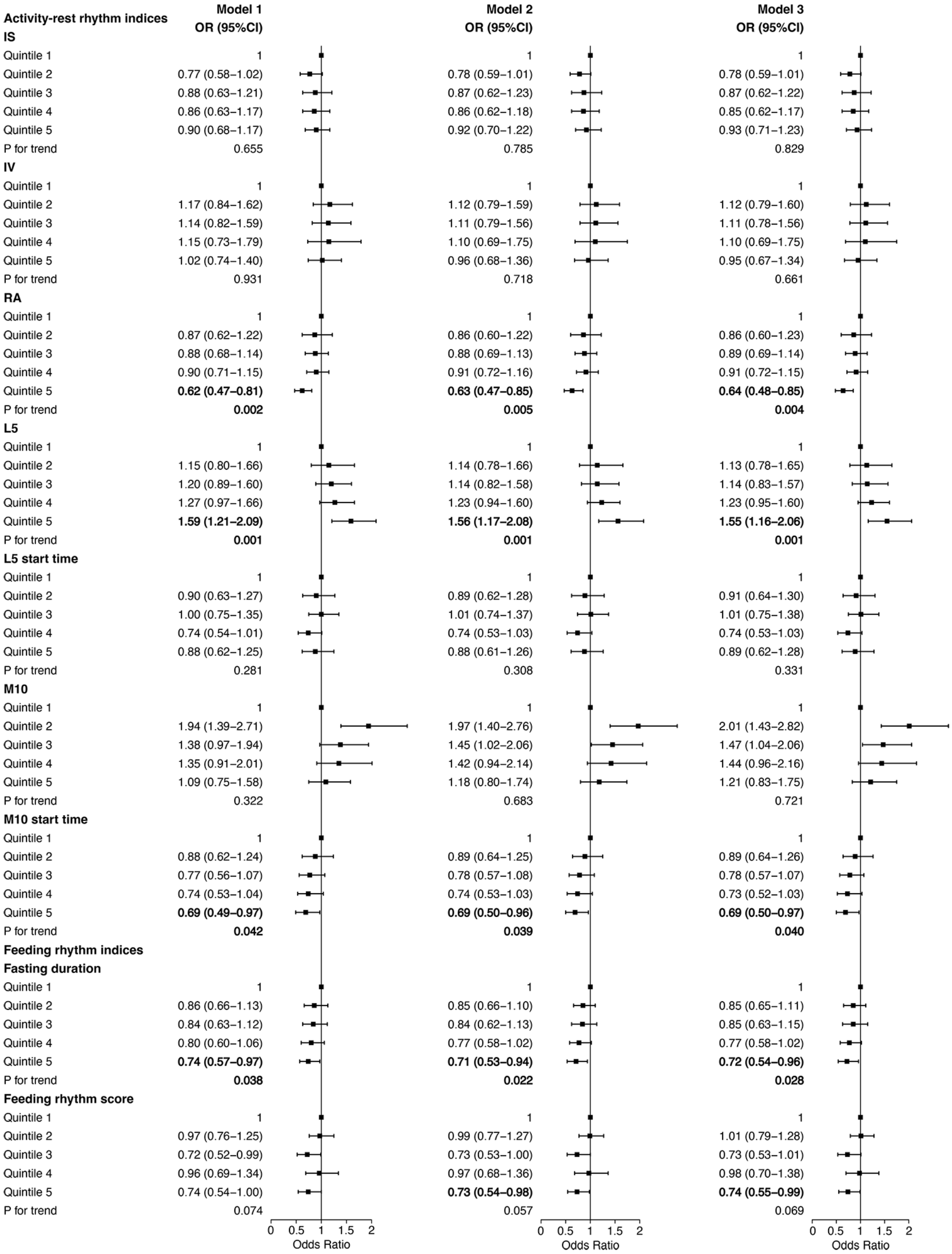

Activity-rest rhythm indices	Model 1 OR (95%CI)	Model 2 OR (95%CI)	Model 3 OR (95%CI)
**IS**			
Quintile 1	1	1	1
Quintile 2	0.77 (0.58–1.02)	0.78 (0.59–1.01)	0.78 (0.59–1.01)
Quintile 3	0.88 (0.63–1.21)	0.87 (0.62–1.23)	0.87 (0.62–1.22)
Quintile 4	0.86 (0.63–1.17)	0.86 (0.62–1.18)	0.85 (0.62–1.17)
Quintile 5	0.90 (0.68–1.17)	0.92 (0.70–1.22)	0.93 (0.71–1.23)
P for trend	0.655	0.785	0.829
**IV**			
Quintile 1	1	1	1
Quintile 2	1.17 (0.84–1.62)	1.12 (0.79–1.59)	1.12 (0.79–1.60)
Quintile 3	1.14 (0.82–1.59)	1.11 (0.79–1.56)	1.11 (0.78–1.56)
Quintile 4	1.15 (0.73–1.79)	1.10 (0.69–1.75)	1.10 (0.69–1.75)
Quintile 5	1.02 (0.74–1.40)	0.96 (0.68–1.36)	0.95 (0.67–1.34)
P for trend	0.931	0.718	0.661
**RA**			
Quintile 1	1	1	1
Quintile 2	0.87 (0.62–1.22)	0.86 (0.60–1.22)	0.86 (0.60–1.23)
Quintile 3	0.88 (0.68–1.14)	0.88 (0.69–1.13)	0.89 (0.69–1.14)
Quintile 4	0.90 (0.71–1.15)	0.91 (0.72–1.16)	0.91 (0.72–1.15)
Quintile 5	**0.62 (0.47–0.81)**	**0.63 (0.47–0.85)**	**0.64 (0.48–0.85)**
P for trend	**0.002**	**0.005**	**0.004**
**L5**			
Quintile 1	1	1	1
Quintile 2	1.15 (0.80–1.66)	1.14 (0.78–1.66)	1.13 (0.78–1.65)
Quintile 3	1.20 (0.89–1.60)	1.14 (0.82–1.58)	1.14 (0.83–1.57)
Quintile 4	1.27 (0.97–1.66)	1.23 (0.94–1.60)	1.23 (0.95–1.60)
Quintile 5	**1.59 (1.21–2.09)**	**1.56 (1.17–2.08)**	**1.55 (1.16–2.06)**
P for trend	**0.001**	**0.001**	**0.001**
**L5 start time**			
Quintile 1	1	1	1
Quintile 2	0.90 (0.63–1.27)	0.89 (0.62–1.28)	0.91 (0.64–1.30)
Quintile 3	1.00 (0.75–1.35)	1.01 (0.74–1.37)	1.01 (0.75–1.38)
Quintile 4	0.74 (0.54–1.01)	0.74 (0.53–1.03)	0.74 (0.53–1.03)
Quintile 5	0.88 (0.62–1.25)	0.88 (0.61–1.26)	0.89 (0.62–1.28)
P for trend	0.281	0.308	0.331
**M10**			
Quintile 1	1	1	1
Quintile 2	1.94 (1.39–2.71)	1.97 (1.40–2.76)	2.01 (1.43–2.82)
Quintile 3	1.38 (0.97–1.94)	1.45 (1.02–2.06)	1.47 (1.04–2.06)
Quintile 4	1.35 (0.91–2.01)	1.42 (0.94–2.14)	1.44 (0.96–2.16)
Quintile 5	1.09 (0.75–1.58)	1.18 (0.80–1.74)	1.21 (0.83–1.75)
P for trend	0.322	0.683	0.721
**M10 start time**			
Quintile 1	1	1	1
Quintile 2	0.88 (0.62–1.24)	0.89 (0.64–1.25)	0.89 (0.64–1.26)
Quintile 3	0.77 (0.56–1.07)	0.78 (0.57–1.08)	0.78 (0.57–1.07)
Quintile 4	0.74 (0.53–1.04)	0.74 (0.53–1.03)	0.73 (0.52–1.03)
Quintile 5	**0.69 (0.49–0.97)**	**0.69 (0.50–0.96)**	**0.69 (0.50–0.97)**
P for trend	**0.042**	**0.039**	**0.040**
**Feeding rhythm indices**			
**Fasting duration**			
Quintile 1	1	1	1
Quintile 2	0.86 (0.66–1.13)	0.85 (0.66–1.10)	0.85 (0.65–1.11)
Quintile 3	0.84 (0.63–1.12)	0.84 (0.62–1.13)	0.85 (0.63–1.15)
Quintile 4	0.80 (0.60–1.06)	0.77 (0.58–1.02)	0.77 (0.58–1.02)
Quintile 5	**0.74 (0.57–0.97)**	**0.71 (0.53–0.94)**	**0.72 (0.54–0.96)**
P for trend	**0.038**	**0.022**	**0.028**
**Feeding rhythm score**			
Quintile 1	1	1	1
Quintile 2	0.97 (0.76–1.25)	0.99 (0.77–1.27)	1.01 (0.79–1.28)
Quintile 3	0.72 (0.52–0.99)	0.73 (0.53–1.00)	0.73 (0.53–1.01)
Quintile 4	0.96 (0.69–1.34)	0.97 (0.68–1.36)	0.98 (0.70–1.38)
Quintile 5	0.74 (0.54–1.00)	**0.73 (0.54–0.98)**	**0.74 (0.55–0.99)**
P for trend	0.074	0.057	0.069

Figure S4. Association of 24 h behavior rhythm indices with NAFLD among current drinkers with over-weight/obesity. Model 1 was adjusted for age, gender and race; Model 2 was additionally adjusted for BMI, education level, income, current smoking status, regular exercise and energy; Model 3 was further adjusted for self-reported diabetes, hypertension and hypercholesterolemia. BMI, body mass index; Q, quartile; IS, interdaily stability; IV, intradaily variability; RA, relative amplitude; L5, av-erage activity of the least active continuous 5 h period; M10, average activity of the most active continuous 10 h period.

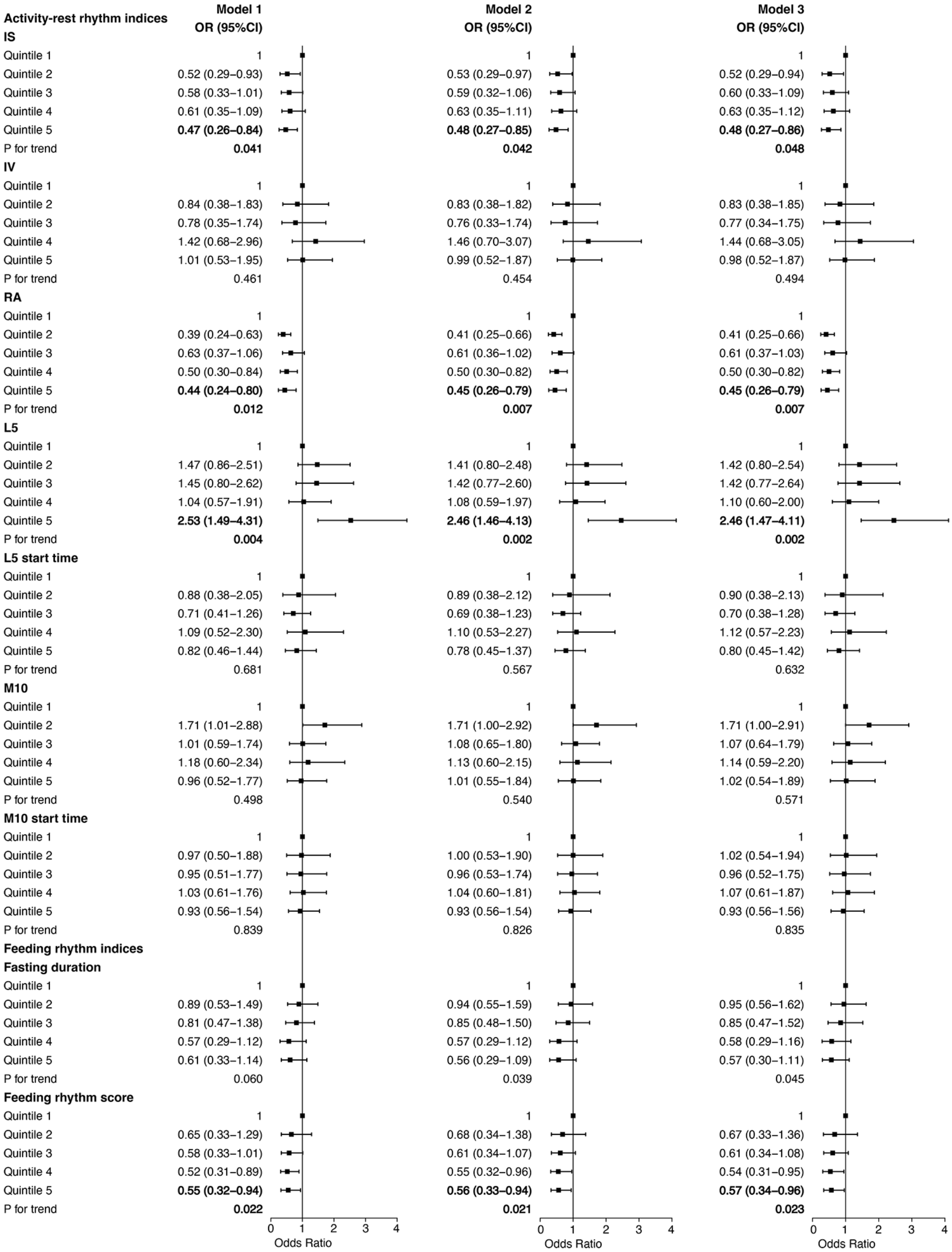

Figure S5. Association of 24 h behavior rhythm indices with NAFLD among current smokers with over-weight/obesity. Model 1 was adjusted for age, gender and race; Model 2 was additionally adjusted for BMI, education level, income, current drinking status, regular exercise and energy; Model 3 was further adjusted for self-reported diabetes, hypertension and hypercholesterolemia. BMI, body mass index; Q, quartile; IS, interdaily stability; IV, intradaily variability; RA, relative amplitude; L5, av-erage activity of the least active continuous 5 h period; M10, average activity of the most active continuous 10 h period.

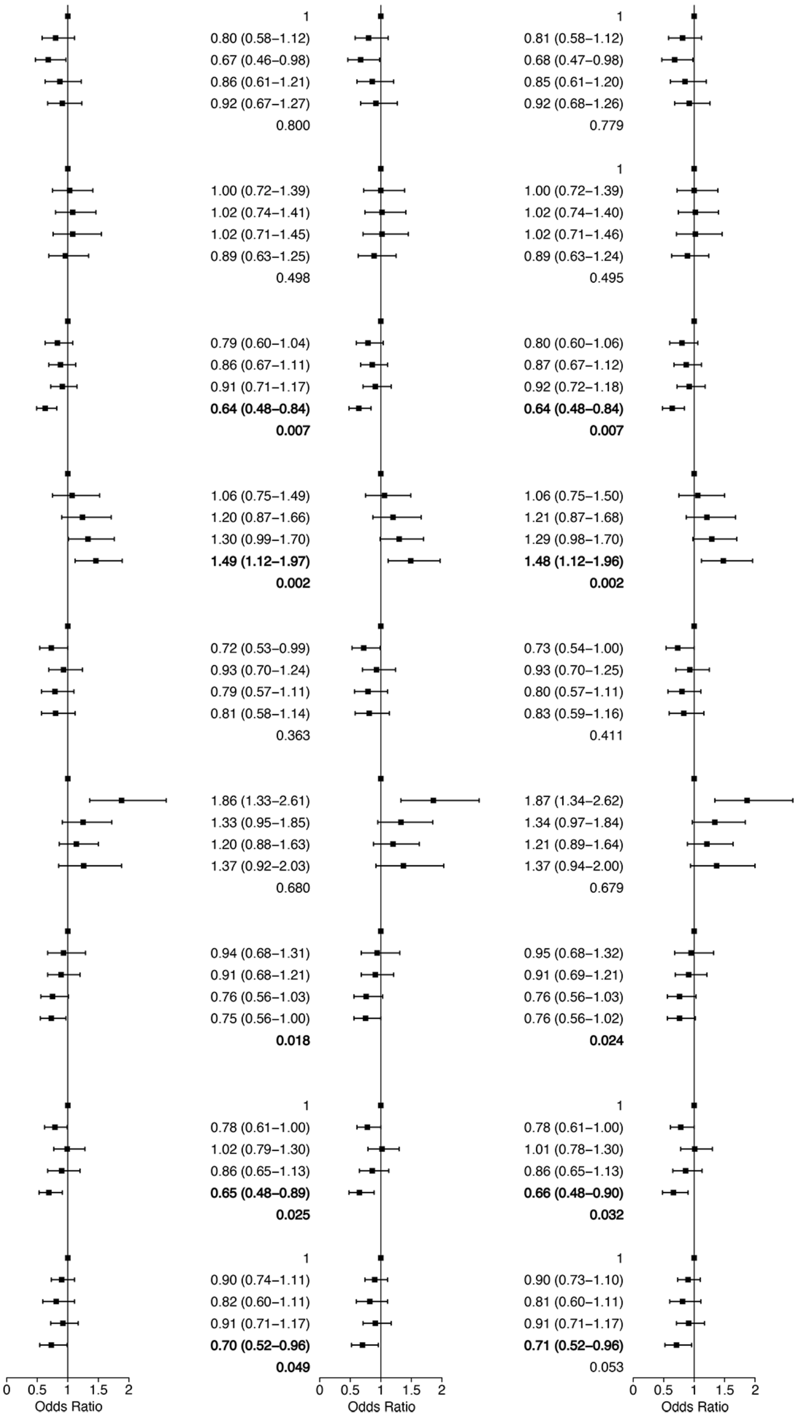

Activity-rest rhythm indices	Model 1 OR (95%CI)	Model 2 OR (95%CI)	Model 3 OR (95%CI)
**IS**			
Quintile 1	1	1	1
Quintile 2	0.80 (0.58–1.11)	0.80 (0.58–1.12)	0.81 (0.58–1.12)
Quintile 3	0.68 (0.47–0.97)	0.67 (0.46–0.98)	0.68 (0.47–0.98)
Quintile 4	0.87 (0.63–1.22)	0.86 (0.61–1.21)	0.85 (0.61–1.20)
Quintile 5	0.91 (0.67–1.23)	0.92 (0.67–1.27)	0.92 (0.68–1.26)
P for trend	0.733	0.800	0.779
**IV**			
Quintile 1	1		1
Quintile 2	1.03 (0.75–1.41)	1.00 (0.72–1.39)	1.00 (0.72–1.39)
Quintile 3	1.08 (0.80–1.46)	1.02 (0.74–1.41)	1.02 (0.74–1.40)
Quintile 4	1.08 (0.76–1.55)	1.02 (0.71–1.45)	1.02 (0.71–1.46)
Quintile 5	0.96 (0.69–1.34)	0.89 (0.63–1.25)	0.89 (0.63–1.24)
P for trend	0.875	0.498	0.495
**RA**			
Quintile 1	1		
Quintile 2	0.83 (0.63–1.08)	0.79 (0.60–1.04)	0.80 (0.60–1.06)
Quintile 3	0.88 (0.69–1.13)	0.86 (0.67–1.11)	0.87 (0.67–1.12)
Quintile 4	0.91 (0.72–1.15)	0.91 (0.71–1.17)	0.92 (0.72–1.18)
Quintile 5	**0.63 (0.49–0.82)**	**0.64 (0.48–0.84)**	**0.64 (0.48–0.84)**
P for trend	**0.002**	**0.007**	**0.007**
**L5**			
Quintile 1	1		
Quintile 2	1.07 (0.75–1.52)	1.06 (0.75–1.49)	1.06 (0.75–1.50)
Quintile 3	1.24 (0.90–1.71)	1.20 (0.87–1.66)	1.21 (0.87–1.68)
Quintile 4	1.33 (1.01–1.76)	1.30 (0.99–1.70)	1.29 (0.98–1.70)
Quintile 5	**1.46 (1.12–1.89)**	**1.49 (1.12–1.97)**	**1.48 (1.12–1.96)**
P for trend	**0.002**	**0.002**	**0.002**
**L5 start time**			
Quintile 1	1		
Quintile 2	0.73 (0.54–1.00)	0.72 (0.53–0.99)	0.73 (0.54–1.00)
Quintile 3	0.93 (0.69–1.24)	0.93 (0.70–1.24)	0.93 (0.70–1.25)
Quintile 4	0.79 (0.57–1.10)	0.79 (0.57–1.11)	0.80 (0.57–1.11)
Quintile 5	0.80 (0.57–1.12)	0.81 (0.58–1.14)	0.83 (0.59–1.16)
P for trend	0.295	0.363	0.411
**M10**			
Quintile 1	1		
Quintile 2	1.88 (1.36–2.61)	1.86 (1.33–2.61)	1.87 (1.34–2.62)
Quintile 3	1.25 (0.91–1.72)	1.33 (0.95–1.85)	1.34 (0.97–1.84)
Quintile 4	1.14 (0.86–1.50)	1.20 (0.88–1.63)	1.21 (0.89–1.64)
Quintile 5	1.26 (0.85–1.88)	1.37 (0.92–2.03)	1.37 (0.94–2.00)
P for trend	0.852	0.680	0.679
**M10 start time**			
Quintile 1	1		
Quintile 2	0.93 (0.67–1.29)	0.94 (0.68–1.31)	0.95 (0.68–1.32)
Quintile 3	0.89 (0.67–1.20)	0.91 (0.68–1.21)	0.91 (0.69–1.21)
Quintile 4	0.75 (0.56–1.01)	0.76 (0.56–1.03)	0.76 (0.56–1.03)
Quintile 5	**0.73 (0.55–0.97)**	0.75 (0.56–1.00)	0.76 (0.56–1.02)
P for trend	**0.011**	**0.018**	**0.024**
**Feeding rhythm indices**			
**Fasting time**			
Quintile 1	1	1	1
Quintile 2	0.79 (0.62–0.99)	0.78 (0.61–1.00)	0.78 (0.61–1.00)
Quintile 3	0.99 (0.77–1.28)	1.02 (0.79–1.30)	1.01 (0.78–1.30)
Quintile 4	0.90 (0.67–1.20)	0.86 (0.65–1.13)	0.86 (0.65–1.13)
Quintile 5	**0.69 (0.53–0.91)**	**0.65 (0.48–0.89)**	**0.66 (0.48–0.90)**
P for trend	0.055	**0.025**	**0.032**
**Feeding rhythm score**			
Quintile 1	1	1	1
Quintile 2	0.90 (0.73–1.11)	0.90 (0.74–1.11)	0.90 (0.73–1.10)
Quintile 3	0.81 (0.59–1.11)	0.82 (0.60–1.11)	0.81 (0.60–1.11)
Quintile 4	0.92 (0.72–1.17)	0.91 (0.71–1.17)	0.91 (0.71–1.17)
Quintile 5	**0.73 (0.54–0.99)**	**0.70 (0.52–0.96)**	**0.71 (0.52–0.96)**
P for trend	0.067	**0.049**	0.053

Figure S6. Association of 24 h behavior rhythm indices with NAFLD among participants with over-weight/obesity and non-regular exercise. Model 1 was adjusted for age, gender and race; Model 2 was additionally adjusted for BMI, education level, income, current smoking status, current drinking status and energy; Model 3 was further adjusted for self-reported diabetes, hypertension and hypercholesterolemia. BMI, body mass index; Q, quartile; IS, interdaily stability; IV, intradaily variability; RA, relative amplitude; L5, average activity of the least active continuous 5 h period; M10, average activity of the most active continuous 10 h period.
